# The Initial Response to COVID-19 Disruptions for Older People with HIV in Ukraine

**DOI:** 10.3390/geriatrics7060138

**Published:** 2022-12-06

**Authors:** Julia Rozanova, Katherine M. Rich, Frederick L. Altice, Sheela V. Shenoi, Irina Zaviryukha, Tetiana Kiriazova, Elmira Mamedova, Oleksandr Shipunov, Volodymyr Yariy, Alexandra Deac, Oleksandr Zeziulin

**Affiliations:** 1 Section of Infectious Diseases, Yale University School of Medicine, New Haven, CT 06510, USA; 2 Centre for Interdisciplinary Research on AIDS (CIRA), Yale University School of Public Health, New Haven, CT 06510, USA; 3 Department of Health Service and Population Research, King’s College London, London SE5 8AF, UK; 4 Harvard Medical School, Boston, MA 02115, USA; 5 Centre of Excellence of Research in AIDS (CERiA), University of Malaya, Kuala Lumpur 59990, Malaysia; 6 Division of Epidemiology of Microbial Diseases, Yale University School of Public Health, New Haven, CT 06510, USA; 7 European Institute of Public Health Policy, 04123 Kyiv, Ukraine; 8 Kyiv AIDS Centre, 03115 Kyiv, Ukraine; 9 Kyiv Sociotherapy Addiction Treatment Clinic, 03039 Kyiv, Ukraine

**Keywords:** older adults with HIV, COVID-19 lockdown, HIV care, substance use, depression and anxiety symptoms, Ukraine

## Abstract

Ukraine imposed a COVID-19 lockdown in March 2020. From April to June 2020, we surveyed 123 older people with HIV (OPWH) by phone to assess their mental health, engagement in HIV and other healthcare, and substance use using standardised scales. Variables of key interest were symptoms of depression and symptoms of anxiety. Univariate and multivariable Firth logistic regression models were built to assess factors associated with: (1) symptoms of depression, and (2) symptoms of anxiety. Findings indicated high suicidal ideation (10.6%); 45.5% met the screening criteria for moderate to severe depression; and 35.0% met the criteria for generalised anxiety disorder (GAD). Independent correlates of having moderate to severe depression included being female (AOR: 2.83, 95%CI = 1.19–7.05), having concerns about potential barriers to HIV treatment (AOR: 8.90, 95%CI = 1.31–104.94), and active drug use (AOR: 34.53, 95%CI = 3.02–4885.85). Being female (AOR: 5.30, 95%CI = 2.16–14.30) and having concerns about potential barriers to HIV treatment (AOR: 5.33, 95%CI = 1.22–28.45) were independently correlated with GAD, and over half (58.5%) were willing to provide peer support to other OPWH. These results highlight the impact of the COVID-19 restrictions in Ukraine on mental health for OPWH and support the need to screen for psychiatric and substance use disorders, potentially using telehealth strategies.

## 1. Introduction

The unfolding of the COVID-19 pandemic imposed significant changes on how people live, work, access healthcare support, receive treatment, and interact with each other, with long-lasting negative outcomes on individuals’ physical and mental health [1]. A broad range of evidence indicates that older adults are especially vulnerable to more severe consequences of COVID-19, with a heightened risk for hospitalisation and death [2,3]. For example, a study conducted in Poland during the second wave of COVID-19 investigated the prevalence of common mental health disorders among the elderly after accounting for sociodemographic variables and chronic diseases [4]. The authors recruited 221 individuals (47.51% females and 52.49% males) aged 60+ to assess the anxiety, depression, irritability, and loneliness rates among this vulnerable group. Their results suggest that the experience of a comorbid disease was associated with significantly higher levels of common mental health disorders (i.e., depression, anxiety, and irritability), an increased number of deaths, and having a sense of loneliness and disconnection, with females reporting higher anxiety levels compared to male counterparts. However, the survey was conducted online, which may have excluded even more vulnerable elderly, such as those living alone in remote settings or those unable or unwilling to have technical access. The results highlight the need to enable special care for this vulnerable population, with tailored interventions to account for adverse psychosocial influences and chronic diseases. Also, little is known about how they responded to an impending COVID-19 outbreak as governments and healthcare systems imposed lockdowns to mitigate community transmission of the virus. Older adults, especially those with underlying health conditions, received more explicit advice to shelter in place for extended periods of time, raising concerns about how social isolation may affect their well-being [5,6,7]. COVID-19 lockdown effects on older adults’ mental and physical health have been identified as a high research priority [8,9]. However, early evidence indicates that older adults appeared to have better mental health outcomes during the first months of the COVID-19 pandemic outbreak than their counterparts [10]. In contrast, more recent research suggests that prolonged restrictions, long-COVID-19, and limited access to healthcare services worsened the mental health outcomes among older people [11,12], entailing suicidal ideation and other psychiatric illnesses [13], with long-term impacts on prevention efforts and treatment pathways [14,15].

Empirical evidence about the experiences of older people with HIV (OPWH) is scarce, especially in low- and middle-income countries (LMIC) [16], many of which have experienced cyclical guidance for lockdowns through late 2021 to mitigate spikes in COVID-19 infections. Even though the risk of dying of COVID-19 complications among OPWH was considerably increased compared to the general population [17,18], and loneliness and financial insecurity were predictors of psychological distress [19], available studies are limited to hospitalised cases, which may not be relevant for OPWH living in the community. The first lockdown, often in the absence of spikes in COVID-19 cases, provides a critical lens to view the response of OPWH in LMICs to address their unique challenges in future healthcare delivery and to learn to mitigate the harms of social isolation and disengagement in care.

Since 2010, the Eastern Europe and Central Asia (EECA) region has had the most significant increase in HIV incidence and mortality, which remains unabated [20]. Ukraine, an emblematic LMIC in EECA, has over 360,000 people with HIV (PWH), with a 1% prevalence in the adult population [21]. About a quarter of PWH in Ukraine are OPWH, defined as those aged ≥50 years [22,23,24,25]. While Ukraine has made significant strides in HIV care for key populations, including people who inject drugs, men who have sex with men, and persons engaged in commercial sex work, OPWH have not been a target priority population [26], with emerging challenges to the continuity of care during COVID-19 [27].

On March 18, 2020, within a week after registering the first COVID-19 infection in Ukraine, the government introduced a lockdown [28]; the first cases were remote from the country’s capital, Kyiv. HIV clinics nationally urged patients to shelter in place, adhere to HIV treatment, and observe public health authorities’ recommendations. From March to May 2020, countries, including Ukraine [29], experienced breaches in antiretroviral therapy (ART) supply chains, reduction or complete suspension of in-person visits, and some loss of frontline clinical staff, on whom OPWH often rely for social support [28]. Although existing research draws mostly from high-income countries where HIV prevalence among older adults is lower than that of Ukraine’s [18,30,31,32], it mirrors fears that OPWH in Ukraine likewise exhibited increased vulnerability during the early weeks of the COVID-19 pandemic and substantiates concerns that may drive worsening mental health. Also, while evidence indicated the benefits of the buffering effect of peer support on mental health outcomes during the COVID-19 pandemic [33], to the best of our knowledge, there is scarce research on the possible protective effects of peer support during COVID-19 (or other crises) including older adults with HIV in Ukraine.

To address the status of OPWH during the initial lockdown in response to COVID-19, we conducted a cross-sectional phone survey among OPWH, gathering information on lockdown experiences. Our findings will help guide mental health interventions under conditions of adversity, including future COVID-19 restrictions, given low vaccination coverage in LMIC, and continuing political uncertainty, conflict, and economic difficulties, especially in Ukraine.

## 2. Methods

### 2.1. Ethics Statement

This study was approved by the institutional review boards at Yale University (IRB Protocol #2000022278) and European Institute on Public Health Policy in Ukraine (IRB Protocol #2017-021-08).

### 2.2. Study Participants and Procedures

As an immediate response to the lockdown, we met with our community working group (CWG) of key stakeholders, including OPWH, psychologists, and doctors specialising in HIV care and/or addiction, to create a survey instrument. After IRB approval, participants were recruited from May 08, 2020, through June 14, 2020, from the two largest HIV care providers in Kyiv. Namely, Kyiv City AIDS Center, which serves 14,000 PWH annually (1289 being OPWH) and “Sociotherapy”, an addiction treatment clinic that integrates HIV services for 939 PWH with a history of addiction (112 of them being OPWH). Participants were recruited by phone by trained staff (psychologists or social workers with officially authorised access to clinic patients’ database) using convenience sampling.

Eligibility criteria were: 50 years of age or older; currently registered for HIV care at one of the participating clinics; and willing to participate in a phone survey. Clinical staff approached 147 potential participants; 125 OPWH were successfully reached, and 123 agreed to complete the 40 (range: 30–60) minute survey (target sample size was 100, to provide immediate feedback to community stakeholders). Participants completing the survey were paid 200 Ukrainian hryvnias (~8 USD) as either telephone credit or bank transfer (participant’s choice). Participants who reported high levels of distress (i.e., suicidal ideation) in the past two weeks or needed medical advice were reported to a clinician using the standard protocol; all others were provided with the number of a mental health hotline for their future use. The survey was conducted in Russian or Ukrainian (per participant’s choice).

Variables and Scales: Literature review informed variable selection [34,35,36]. We collected information across six domains: (1) demographic and general characteristics, (2) response to COVID-19 (exposures, isolation, self-reported stressors), (3) HIV care (adherence assessed by a 7-day recall scale that has previously been used in Ukraine) [37], (4) psychiatric and substance use disorders [38,39] including the Generalized Anxiety Disorder-7 scale (GAD-7) and Patient Health Questionnaire-9 (PHQ-9) scales, (5) psychosocial characteristics, and (6) factors related to chronic health conditions (see Table 1 below).

Patients were classified as having a drug or alcohol use disorder either if they self-reported a diagnosis or if they were recruited from an addiction treatment clinic. Psychosocial characteristics included social support based on a 10-item version of the scale used in Ukraine in previous studies [40] and resilience using the Brief Resilience Scale (BRS) [41]. The phrasing of the COVID-19-related questions was informed by consultations with US and Ukrainian experts in geriatric and mental health research who reviewed the final version of the survey instrument and made recommendations to ease surveying participants by phone. See Table S1 for a full description of the measures and scales used. Additionally, participants were asked about HIV care support preferences and whether they would be willing to provide peer support to other OPWH.

Development of survey: Initial development of the survey was completed by the authorship team in English. After drafting, the survey was translated by members of the study team into Ukrainian. Following initial development, we piloted the survey with the interviewers and 3 OPWH consultants. OPWH consultants provided feedback to our research team and to the interviewers on question wording and length of the survey. After incorporating recommended changes, the instrument was finalized, and survey collection began with our recruited sample.

### 2.3. Dependent Variables

Symptoms of depression and generalised anxiety disorder (GAD) were the primary outcomes and were categorised based on the screening criteria of symptoms. We defined symptoms of depression as a score ≥5 on the PHQ-9 scale [42]. The PHQ-9 consists of 9 questions with a possible score of 0 to 27, with higher scores correlated with increased symptoms of depression. Similarly, we defined symptoms of GAD as having a score of ≥5 on the GAD-7 [43]. The GAD-7 scale consists of 7 questions with a possible score ranging from 0 to 21. The Ministry of Health of Ukraine recommends both the PHQ-9 and GAD-7 scales for screening purposes [44,45]; the adapted and validated versions of GAD-7 and PHQ-9 comprised forward and backward translation, specialist debriefing and final proofreading [46,47], and these scales have been used previously among PWH in Ukraine [48,49]. We evaluated lower thresholds of depression and anxiety symptoms as clinically meaningful, as monitoring is warranted among older patients with complex needs with mild symptoms [42,50,51], and to understand the prevalence and correlates of any anxiety and depression during COVID-19.

### 2.4. Analytic Strategy

Statistical analysis was performed with de-identified data and all the calculations were conducted using R version 4.0.0 (R Core Team, 2020; Vienna: Austria). We described participant characteristics using means and standard deviations for continuous variables and frequencies and percentages for categorical variables. Participant characteristics were compared by depression and anxiety classification using Student’s t-test for continuous variables and chi-square or Fisher’s exact tests for categorical variables. Statistical significance was defined at *p* < 0.05.

Multivariable regression analyses were conducted for the two outcomes: (1) depressive symptoms, as assessed with the PHQ-9 scale; and (2) GAD symptoms, as assessed with the GAD-7 scale. All variables with *p* < 0.10 in bivariate testing were entered into the multivariable model. Collinearity of independent variables was assessed using the variance inflation factor (VIF). A Firth logistic regression model was used due to the complete separation of variables and for sample size considerations [52,53]. The relationship between depressive and GAD symptoms was assessed using Pearson correlation.

## 3. Results

Among the 123 participants, 73 (59.3%) were recruited from Kyiv AIDS Center. Table 1 provides the descriptive statistics for OPWH, with half (n = 61, 49.6%) being women and the mean age being 55.3 years (SD = 6.80; median = 54; IQR = 50.0–59.5). All participants had completed high school, with 58.5% having completed vocational secondary education and 30.9% having a higher education. Forty percent (n = 52, 40.7%) had a substance use disorder, including alcohol use disorder. During the COVID-19 lockdown, a quarter of the participants (n = 32, 26.0%) were isolating at home alone, and nearly half (n = 55, 44.7%) were isolating with family. A third of the sample (n = 36, 29.3%) were not isolating. While only 7 (5.7%) reported being tested for COVID-19, none reported a positive result. One-fifth (n = 22, 17.9%) reported having <1 social support during lockdown (i.e., someone to trust and could be asked for help).

Nearly half of the participants (n = 56, 45.5%) had symptoms of depression, and 35.0% (n = 43) had symptoms of anxiety (Table 1). Thirty-four (27.6%) participants had symptoms of both anxiety and depression, yet only seven (5.7%) reported that their mental health had worsened during COVID-19. Thirteen (10.6%) individuals reported suicidal ideation during the last two weeks, of which ten had symptoms of both depression and anxiety. A majority of the sample (n = 86, 69.9%) reported at least one comorbidity in addition to HIV. Of the 86 individuals with chronic conditions, 17 (19.8%) reported taking none or almost none of their medications for their chronic condition in the last 7 days.

Regarding HIV care, almost all OPWH (n = 117, 95.1%) had been previously prescribed ART, with 112 patients (91.1%) currently receiving ART, and five individuals disengaged from ART. Of individuals prescribed ART, seven (6.3%) reported missing a dose in the past 7 days. Less than half of those prescribed ART (n = 47, 42.0%) reported having an individual to assist with HIV treatment during the lockdown. Of these 47 participants, 34 (72.3%) had a clinic staff person (i.e., doctor, nurse, psychologist, or social worker) providing assistance and 23 (48.8%) reported having a friend or a family member providing HIV treatment assistance.

When asked about preferences regarding HIV peer support, a majority (n = 72, 58.5%) reported a willingness to provide HIV peer support to other OPWH (see Table 2 below). Many OPWH preferred that the peer supporter was also HIV-positive (n = 59, 48.0%). Some participants preferred that a “peer supporter” was the same age (n = 7, 5.7%), same gender (n = 14, 11.4%), and/or same sexual orientation (n = 4, 3.3%). Many preferred that the individual had expertise in medicine (n = 53, 43.1%).

### Univariate and Multivariable Modelling of Depression and GAD

Univariate analysis identified several factors that significantly differed when stratifying by depression symptoms and anxiety symptoms. Full results are presented in Tables S3 and S4 for depression and anxiety, respectively. PHQ-9 and GAD-7 scores had high correlation, with a Pearson correlation coefficient of 0.75. Accordingly, the variables were not included as independent variables.

In multivariable modelling, the independent correlates of depression were female sex (aOR = 2.83; 95% CI  =  1.19–7.05), reporting barriers to HIV treatment as a stressor during lockdown (aOR = 8.90; 95% CI  =  1.31–104.94), and reporting illicit drug use during lockdown (aOR = 34.53; 95% CI  =  3.02–4885.85). Full multivariable results are presented in Table 3. Multicollinearity was not observed, with all VIFs <2.3.

Being female (aOR = 5.30; 95% CI = 2.16–14.30) and reporting barriers to HIV treatment as a stressor of lockdown (aOR = 5.33; 95% CI = 1.22–28.45) was associated with reporting anxiety. In addition, having an individual identified as providing HIV support was associated with increased anxiety (aOR = 2.68; 95% CI = 1.07–6.95). Multicollinearity was not observed, with all VIFs <2.0. Full multivariable results are presented in Table 4.

## 4. Discussion

During the initial lockdown in response to COVID-19, there was a high prevalence of moderate to severe depression and GAD among OPWH. Findings from OPWH in Ukraine had a relatively higher prevalence than reported elsewhere, mostly in high-income settings, and to a lesser extent, in younger persons (for example, in China, GAD prevalence in a younger urban adult sample of people with HIV was 40.3% [50] versus 35% in our study). A high proportion of participants with a SUD diagnosis in our sample may explain the high prevalence of depression. However, other studies also found higher depression rates among people who inject drugs in Ukraine than in countries such as Vietnam and Indonesia [54].

In comparison to our findings, a US study found a much lower prevalence of depression and anxiety symptoms in older adults, in general, during the COVID-19 pandemic: 5.8% and 6.2%, respectively, among those 65 and older, and 14.1% and 16.4% among those 45 to 64, respectively [8]. While half of our OPWH participants met screening criteria for moderate to severe depression during the first COVID-19 lockdown, previous assessments of depression among Ukrainian adults 50 years and older were much lower (14.4% for women and 7.1% for men), although only major depressive episodes were reported [55]. At a minimum, these findings suggest that expanded mental health care is needed for OPWH, particularly among women relative to men. However, this finding that women experience more GAD and depression than their male counterparts may reflect similar findings elsewhere in LMIC settings before COVID [56,57]. Additional contributors to elevated mental health concerns may relate to findings in Ukraine that suggest that HIV stigma contributes to worse mental health [58], and because women reflect relatively lower levels of HIV than their male counterparts, their mental health may be affected by minority stress [59,60,61].

Findings here differed from reports in high-income settings during COVID-19, where women were more likely to self-report exacerbations in their mental health [62]. However, women and men in this study rarely reported worsening or new anxiety since the lockdown. Several explanations could support this finding in Ukraine, including that despite the first lockdown, there were not many cases of COVID-19 and the disease was perceived abstractly rather than as a reality. Alternatively, this group of OPWH had so many other challenges in their lives that COVID-19 was just another with which they had to deal. Last, Ukraine’s psychiatric services (and related illnesses) are highly stigmatised. Despite standardised measures suggesting high levels of mental illness, the participants in this sample were not willing to report it and perceived it as a weakness—possibly a vestige of Soviet-style medicine and the legacy of punitive-style treatments for mental illness during that time—a period that all participants lived through into at least early adulthood [63].

OPWH were highly concerned about potential disruptions in their HIV care, which was independently correlated with both moderate to severe depression and GAD. As a chronic disease requiring a lifetime of treatment, it is not surprising that treatment interruptions might result in adverse consequences. Data from the SMART trial suggested that PWH whose ART was disrupted were significantly more likely to experience an adverse consequence, including non-HIV-related diseases, such as myocardial infarction and stroke [64], for which OPWH are generally already at higher risk. Moreover, despite later responses suggesting limited stockouts and HIV providers extending medications to 30 rather than 90 days, having to pick up medications frequently in the setting of a lockdown may have contributed to elevated stress. Last, the health system reforms that have been underway in Ukraine since 2017 resulted in some instability that may have complicated timely HIV services delivery during the lockdown [65].

Unexpectedly, anxiety symptoms were associated with having someone to assist with taking HIV medications. We speculate this may remind OPWH of isolation or their fragile health. This finding is in contrast to a substantial body of pre-COVID-19 literature on the beneficial impacts of social support on HIV care cascade outcomes [66,67]. One interpretation of this finding stems from the Health Behaviour Model for Vulnerable Populations, which suggests that more medical comorbidities may predispose to more healthcare-seeking behaviour [68,69]. OPWH could be more anxious about accessing HIV care during the COVID-19 lockdown and may seek and find more assistance, but a cross-sectional assessment does not allow for establishing causality. Another possible explanation is that assistance with taking ART which includes reminders and control over whether medications have been taken properly, is viewed by older adults as a form of clinical care that has coercive and surveillant elements and is thus quite distinct from social support. This explanation stems from the theory of learned helplessness during depression [70,71]: OPWH may resent constant control and reminders about their HIV care as it suggests they are incapable of managing their medications. Likely, domains where social support is provided, who provides it, and how they do so matter considerably to OPWH, shaping their HIV and mental health outcomes.

Consistent with the pre-COVID-19 literature, we found that active use of illicit drugs was associated with depressive symptoms among OPWH during the COVID-19 lockdown [35]. However, the vast majority of OPWH, including those with an addiction history, reported low levels of alcohol use, which is consistent with other studies, despite earlier hypotheses that lockdown will result in higher alcohol use [8]. It is possible that social support (including by trusted clinicians) to vulnerable OPWH with comorbid addictions has played a significant protective role during the COVID-19 lockdown [27]. Early findings from other global regions [72] also suggest that patients with comorbid SUD and HIV who were stably linked to addiction care had no significant changes in risk behaviours during the COVID-19 lockdown.

That 1 in 10 participants reported suicidal ideation is a very grave finding and reinforces concern about the effect of social isolation during the lockdown on OPWH’s mental health and well-being [8,73]. However, dubbing older adults as “vulnerable” and needing extra support perpetuates the problem [73,74,75]. Instead, emerging research suggests that altruism and making societal contributions play a protective role in older adults’ mental health during the COVID-19 pandemic [5]. This resonates with our finding that a majority of OPWH wished to help somebody like themselves during the lockdown, underscoring that peers may be a particularly meaningful source of support for OPWH during COVID-19, with potential buffering effects on fear of anticipated HIV stigma and sense of isolation.

Future research should comparatively study the mental health effects of COVID-19 and other crises on older adults with HIV, including natural disasters or man-made tragedies such as the war, as well as explore whether interaction with peers can strengthen HIV and mental health outcomes in OPWH during crises [5]. While mitigation strategies for COVID-19-related social isolation may include screening for anxiety and depression during clinical phone check-ins, a comprehensive transition to telehealth calls as well as ensuring uninterrupted supply chains of essential medications such as ART needs to also account for the inclusion of OPWH in mitigation efforts (e.g., peer support text chat groups). Lessons from the COVID-19 pandemic suggest the need for tailored, proactive, and empowering interventions, from the preparedness stage to emergent responses to long-term, sustainable planning of post-crisis relief efforts, and OPWH need to be included in each stage of disaster public health response planning [76,77].

Nevertheless, gaps remain in capturing the plethora of social isolation, the stigma of disclosing the HIV status and a COVID-19-positive test to unfamiliar personnel, and its impact on OPWH’s mental health in community-centred societies such as Ukraine [55].

We recognise some limitations. First, the sample size limited the precision of our models and resulted in wide confidence intervals of our estimates. Second, the cross-sectional design allowed for exploring associations between depressive and anxiety symptoms and other factors but did not establish causality. Also, while we used previously validated scales for our key outcomes, including depression and anxiety, and other variables, including resilience, social support, and alcohol use, these variables have not been validated specifically for OPWH in Ukraine. Thus, it would be advantageous if future research included a validation of the various mental health measures among older adults with HIV in Ukraine. Despite these limitations, our cross-sectional survey findings during the Spring 2020 COVID-19 lockdown provide an important baseline for all future work to longitudinally examine trajectories in the mental health of OPWH, HIV and other healthcare adherence, and substance use, especially as COVID-19 lockdowns were re-introduced in Ukraine (since November 1, 2021) and other countries.

## 5. Conclusions

The first COVID-19 Spring 2020 lockdown in Ukraine was associated with a high and gendered prevalence of depression and anxiety symptoms. Perceived barriers to HIV care and self-reported substance use were also associated with higher levels of depression and anxiety symptoms among OPWH during the lockdown. While the majority of OPWH reported continuing HIV and addiction care during the COVID-19 lockdown, substantial non-adherence to care for conditions other than HIV was reported. Findings highlight the need to consider specific challenges of OPWH, including comorbid physical and mental health conditions during healthcare delivery in the context of the COVID-19 pandemic, and suggest exploring in future studies whether support from other OPWH would strengthen HIV and mental health outcomes. Longitudinal research into short-, mid- and long-term mental health impacts of the COVID-19 pandemic on the mental health and well-being of vulnerable patient groups of older adults must be conducted. In the context of the currently ongoing war in Ukraine, this study may provide a useful baseline for further work on how older adults with HIV cope with their mental (and physical) health.

## Figures and Tables

**Table 1:** Characteristics of older people with HIV (N = 123).

General Characteristics		Total (N = 123)
**Sex**	Female	61 (49.6%)
	Male	62 (50.4%)
**Age**	Mean (SD)	55.3 (6.80)
**Education**	Completed high school	3 (2.4%)
	Attended vocational secondary school	72 (58.5%)
	Some higher education	10 (8.1%)
	Completed higher education	38 (30.9%)
**Smartphone**	Owns a smartphone	90 (73.2%)
**Health**	Chronic condition in addition to HIV	86 (69.9%)
**Response to COVID-19 Lockdown**		
**Self-Isolation**	Yes, by myself	32 (26.0%)
	Yes, with a partner/spouse/family member	55 (44.7%)
	No	36 (29.3%)
**COVID-19 exposure**	Direct exposure	3 (2.4%)
	Family member or friend with COVID-19	11 (8.9%)
**COVID-19 testing**	Received a COVID-19 test	7 (5.7%)
**COVID-19 infection**	Received a positive test	0 (0.0%)
**Personal stressors**	Total # of COVID-19 and lockdown-related stressors (Scale Range: [0–20], mean (SD))	2.58 (1.86)
**HIV treatment stress**	Patient reports stress of possible HIV care disruption	12 (9.8%)
**Economic stress**	Reported any economic worry	51 (41.5%)
	Reported loss of income since the start of the lockdown	28 (22.8%)
**Perceived worsening in the mental health status**	Reported worsening of mental health since the beginning of the lockdown	7 (5.7%)
	Felt more alone since the lockdown	9 (7.3%)
	**HIV Care**	
**Disclosure**	Patient has disclosed HIV status	88 (71.5%)
**Care prior to COVID-19**	Patient registered at an AIDS Centre	120 (97.6%)
	Patient has been prescribed ART ever	117 (95.1%)
	Patient is currently prescribed ART	112 (91.1%)
**ART adherence**	Patient has NOT missed a dose of ART in the past 7 days	110 (89.4%)
**HIV support**	Someone supports the patient’s HIV treatment	47 (38.25)
**Volunteer interest**	Patient is willing to provide peer support	72 (58.5%)
**Drug and Alcohol Use**		
**Drug use**	Illicit drug use during the COVID-19 lockdown	7 (5.7%)
**Addiction**	Diagnosis of substance use and/or alcohol use disorder	52 (42.3%)
**Alcohol use disorder (AUDIT-C)**	No	114 (92.7%)
	Yes	9 (7.3%)
**Psychosocial**		
**Depression**	Depressive symptoms on PHQ-9	56 (45.5%)
**Suicidal ideation**	Reported suicidal ideation	13 (10.6%)
**Anxiety**	Symptoms of generalised anxiety disorder (GAD)	43 (35.0%)
**Social support**	≤1 social supports	22 (17.9%)
**Resilience**	Low level of resilience	44 (35.8%)
	Normal level of resilience	79 (64.2%)

Abbreviations: # = number, ART = antiretroviral therapy, GAD = generalised anxiety disorder, PHQ-9 = patient health questionnaire (9-question scale) to assess depression, SD = standard deviation.

**Table 2:** Peer Support Preferences of OPWH in Kyiv.

Desired Characteristics		n (%)
	HIV positive	59 (48.0%)
	Concordant gender	14 (11.4%)
	Similar age	7 (5.7%)
	Concordant sexual orientation	4 (3.3%)
	Expertise in medicine	53 (43.1%)
**Willingness to provide support**	Yes	72 (58.5%)
	No	51 (41.55)

**Table 3:** Multivariate correlates of depressive symptoms among OPWH in Ukraine (n = 123) ^1^.

General Characteristics		Multivariable aOR (95% CI)	*p*-Value
**Sex**	Female	2.83 (1.19–7.05)	0.018
	Male	Ref	
**Age**	Age of participant	0.96 (0.89–1.03)	0.266
**Response to COVID-19 Lockdown**			
**Total stressors**	Total # of COVID-19 and lockdown-related stressors	0.93 (0.64–1.34)	0.686
**HIV Care**			
**HIV treatment stress**	Patient reports concerns about possible disruptions in HIV care	8.90 (1.31–104.94)	0.024
**HIV support**	Someone supports the patient’s HIV treatment	2.40 (0.99–6.00)	0.054
**Drug and Alcohol Use**			
**Drug use**	Illicit drug use during COVID-19 lockdown	34.53 (3.02–4885.85)	0.002
**Psychosocial**			
**Resilience**	Low level of resilience	1.97 (0.77–5.04)	0.156
	Normal level of resilience	Ref	

Abbreviations: aOR = adjusted odds ratio. ^1^ Variables not listed in Table 1 were not significant in bivariable analysis and not included in Table 2. Full bivariable modelling results are presented in the supplement.

**Table 4:** Multivariate correlates of generalised anxiety disorder symptoms among OPWH in Ukraine (n = 123) ^1^.

General Characteristics		Multivariable aOR (95% CI)	*p*-Value
**Sex**	Female	5.30 (2.16–14.30)	<0.001
	Male	Ref	
**Age**	Age of participant	1.02 (0.95–1.09)	0.537
**Response to COVID-19 Lockdown**			
**Self-Isolation**	Yes, by myself	Ref	0.503
	Yes, with a partner/spouse/family member	0.92 0.33–2.53)	
	No	0.64 (0.18–2.25)	
**Total stressors**	Total # of COVID-19 and lockdown-related stressors	1.02 (0.79–1.30)	0.877
**HIV Care**			
**HIV treatment stress**	Patient reports concerns about possible disruptions in HIV care	5.33 (1.22–28.45)	0.023
**HIV support**	Someone supports the patient’s HIV treatment	2.68 (1.07–6.95)	0.020
**Psychosocial**			
**Resilience**	Low level of resilience	1.91 (0.80–4.63)	0.134
	Normal level of resilience	Ref	

Abbreviations: aOR = adjusted odds ratio. ^1^ Variables not listed in Table 1 were not significant in bivariable analysis and not included in Table 2. Full bivariable modelling results are presented in the supplement.

## Data Availability

Data collected by the team are not available in the public domain. If you wish to collaborate with the team on analyses using these data, please contact the corresponding author.

## Supplementary Materials

The following supporting information can be downloaded at: https://www.mdpi.com/article/10.3390/geriatrics7060138/s1, Table S1: Analysis variable dictionary; Table S2: Full Bivariate and Multivariable Modeling: Correlates of depressive symptoms; Table S3: Full Bivariate and Multivariable Modeling: Correlates of having Generalized Anxiety Disorder; Table S4: Stressors reported by OPWH during first COVID-19 Lockdown in Kyiv; Table S5: Peer Support Preferences of OPWH in Kyiv.
