# Supplementary material for: The Initial Response to COVID-19 Disruptions for Older People with HIV in Ukraine
Source: Geriatrics (Basel). 2022 Dec 6;7(6):138. doi: 10.3390/geriatrics7060138 (PMC9777936; doi:10.3390/geriatrics7060138)
Supplement: Supplementary file 1 [file geriatrics-07-00138-s001.zip › geriatrics-2018064-supplementary.pdf]

Supplementary Tables

Supplementary Table S1. Analysis variable dictionary

	**General Characteristics**
**Gender**	Categorical: Women, Men, Transgender
**Age**	Continuous
**Household**	Categorical: Living alone, living with a partner, living with family members, other
**Education**	Categorical: Did not complete high school, completed high school, attended technical school, attended higher education, completed a degree in higher education
**Smartphone Ownership**	Binary variable regarding smartphone ownership.
	**COVID-19**
**Self-Isolation**	Categorical: Yes, by myself; Yes, with a partner or family member; Not isolating
**Covid-19 Exposure**	1. Binary: Direct Exposure with a known Covid-19 positive individual<br>2. Binary: Family member or friend with known Covid-19
**Specific Stressors during lockdown**	Participants were asked about 20 specific stressors during lockdown, with responses classified as yes or no.
**Total Stressors**	Continuous: Total Number of Stressors (Max 20). Mean and standard deviation were calculated.
**Economic Worry**	Binary: Yes, if a participant reported any sign of economic worry (job loss, price inflation, limited savings, buying food or other necessities)
	**HIV Care**
**Disclosure**	Binary: Patient has disclosed HIV status to someone
**HIV Care prior to Covid-19**	1. Binary: Patient is registered at an AIDS Center<br>2. Binary: Patient has ever been prescribed ART<br>3. Patient is currently prescribed ART
**ART Adherence**	Binary: Participants were asked during how many days in the past week they took ART medication. Patients classified as adherent if they reported missing no doses in last 7 days
**HIV support**	Binary: Patient reports that they have someone who supports HIV treatment (i.e. provides reminders about medication)
**HIV support preferences**	Patients were asked preferences regarding characteristics of those providing HIV care support, including if they preferred individuals with the same HIV status, of a similar age, of the same gender, of the same sexual orientation, and those with medical expertise.
**Willingness to provide HIV peer support**	Binary: Patients reported yes/no if interested in providing peer support to OPWH.
	**Drug and Alcohol Use**
**Drug Use**	Binary: Any reported illicit drug use during Covid-19 lockdown
**Addiction**	Binary: Patient either self-reported a diagnosis of a substance use disorder or an alcohol use disorder OR the patient was recruited as a patient of an addiction treatment clinic
**Alcohol Use (AUDIT-C)**	AUDIT-C: Lower risk drinking (Score <3); Higher risk drinking (Scores ≥ 3)<br>Validation: Bradley et al., 2007; Frank et al., 2008.
	**Psychosocial**

**Social Support**	Patients self-report on the number of individuals they felt like they could trust and turn to for help.
**Resilience (BRS)**	Categorical: The possible score range of the BRS range from 1 to 5. Scores less than 3 classified low resilience, scores between 3 and 4.3 classified normal resilience, and scores between 4.31 and 5 classified higher resilience. (Smith et al., 2018).
	**Chronic Conditions**
**Health**	Binary: Patient has a chronic condition in addition to HIV, including kidney disease, diabetes, heart disease.
**Care during Covid-19**	Regarding chronic conditions care, patients were asked the following yes/no questions:<br>1. Have you had changes to your care routine<br>2. Are you worried about medication shortage<br>3. Have you stopped care due to financial problems<br>4. Can you no longer visit you physician
**Medication Adherence**	Binary: Patients were asked if they had taken all of their pills, most of their pills, about half of their pills, almost none, or none of their pills during the last 7 days. Patients were then categorized in analysis as either taking less than half of pills OR all of most pills. To note: no patients reported about half.

Supplementary Table S2. Full Bivariate and Multivariable Modeling: Correlates of depressive symptoms

General Characteristics		Bivariate OR (95% CI)	Multivariable aOR (95% CI)	p-value
**Sex**	Female	2.59 (1.27 – 5.40)	2.83 (1.19 – 7.05)	0.018
	Male	Ref	Ref	
**Age**	Age of participant	0.97 (0.91 – 1.02)	0.96 (0.89 – 1.03)	0.266
**Education**	Completed High School	Ref		
	Attended Vocational Secondary School	0.14 (0.01 – 1.48)		
	Some Higher Education	0.14 (0.01 – 2.03)		
	Completed Higher Education	0.08 (0.01 – 0.86)		
**Smartphone**	Owns a smartphone	1.00 (0.43 – 2.23)		
**Health**	Chronic condition in addition to HIV	0.97 (0.45 – 2.11)		
**Response to COVID-19 Lockdown**				
**Self-Isolation**	Yes, by myself	Ref		
	Yes, with a partner/spouse/family member	0.96 (0.41 – 2.29)		
	No	0.57 (0.22 – 1.49)		
**Covid-19 Exposure**	Direct Exposure	2.03 (0.26 – 22.71)		
	Family member or friend with COVID-19	1.46 (0.44 – 5.05)		
**Total Stressors**	Total # of COVID-19 and lockdown related stressors	1.35 (1.11 –1.69)	0.93 (0.64 – 1.34)	0.686
**HIV Treatment Stress**	Patient reports concerns about possible disruptions in HIV care	11.21 (2.53 – 105.86)	8.90 (1.31 – 104.94)	0.024
**Economic Stress**	Reported loss of income since start of lockdown	1.61 (0.58 – 4.66)		
**HIV Care**				
**Disclosure**	Patient has disclosed HIV status	1.16 (0.53 – 2.55)		
**Care prior to COVID-19**	Patient registered at an AIDS Center	0.49 (0.04 – 3.81)		
	Patient has been prescribed ART ever	1.54 (0.32 – 9.15)		
	Patient is currently prescribed ART	1.54 (0.33 – 9.15)		
**ART Adherence**	Patient has NOT missed a dose of ART in past 7 days	0.63 (0.11 – 2.97)		
**HIV support**	Someone supports patient's HIV treatment	2.87 (1.38 – 6.13)	2.40 (0.99 – 6.00)	0.054
**Volunteer Interest**	Patient is willing to provide peer support	1.54 (0.75 – 3.19)		
**Drug and Alcohol Use**				
**Drug Use**	Illicit drug use during COVID-19 lockdown	20.45 (2.40 – 2677.81)	34.53 (3.02 – 4885.85)	0.002
**Addiction**	Diagnosis of substance use and/or alcohol use disorder	1.64 (0.72 – 3.81)		
**Alcohol Use Disorder**	No	Ref		
	Yes	2.37 (0.64 – 10.38)		
**Psychosocial**				
**Social Support**	≤1 social supports	0.65 (0.25 – 1.62)		
**Resilience**	Low level of resilience	2.32 (1.11 – 4.96)	1.97 (0.77 – 5.04)	0.156
	Normal level of resilience	Ref	Ref	

Abbreviations: aOR = adjusted odds ratio, ART=antiretroviral therapy, SD=standard deviation

Supplementary Table S3. Full Bivariate and Multivariable Modeling: Correlates of having Generalized Anxiety Disorder

General Characteristics		Bivariate OR (95% CI)	Multivariable aOR (95% CI)	p-value
**Sex**	Female	4.93 (2.25 – 11.47)	5.30 (2.16 – 14.30)	<0.001
	Male	Ref	Ref	
**Age**	Age of participant	1.03 (0.97 – 1.08)	1.02 (0.95 – 1.09)	0.537
**Education**	Completed High School	Ref		
	Attended Vocational Secondary School	0.89 (0.11 – 10.16)		
	Some Higher Education	0.49 (0.04 – 7.21)	--	
	Completed Higher Education	1.10 (0.13 – 12.86)		
**Smartphone**	Owns a smartphone	1.30 (0.57 – 3.12)		
**Health**	Chronic condition in addition to HIV	1.16 (0.52 – 2.65)		
**Self-Isolation**	Yes, by myself	Ref	Ref	
	Yes, with a partner/spouse/family member	0.92 (0.39 – 2.22)	0.92 0.33 – 2.53)	0.503
	No	0.27 (0.08 – 0.78)	0.64 (0.18 – 2.25)	
**Covid-19 Exposure**	Direct Exposure	1.11 (0.10 – 8.60)		
	Family member or friend with Covid-19	0.74 (0.17 – 2.55)		
**Total Stressors**	Total # of COVID-19 and lockdown related stressors	1.27 (1.04 – 1.56)	1.02 (0.79 – 1.30)	0.877
**HIV Treatment Stress**	Patient reports concerns about possible disruptions in HIV care	3.07 (1.44 – 6.69)	5.33 (1.22 – 28.45)	0.023
**Economic Stress**	Reported loss of income since start of lockdown	1.55 (0.53 – 4.36)		
**Disclosure**	Patient has disclosed HIV status	1.47 (0.65 – 3.49)		
**Care prior to Covid-19**	Patient registered at an AIDS Center	0.31 (0.03 – 2.43)		
	Patient has been prescribed ART ever	0.52 (0.11 – 2.57)		
	Patient is currently prescribed ART	1.61 (0.29 – 16.49)		
**ART Adherence**	Patient has NOT missed a dose of ART in past 7d	0.63 (0.11 – 2.97)		
**HIV support**	Has someone to help support patient with their HIV treatment	3.07 (1.44 – 6.69)	2.68 (1.07 – 6.95)	0.020
**Volunteer Interest**	Patient is willing to provide peer support	1.30 (0.62 – 2.79)		
**Drug Use**	Illicit drug use during Covid-19 lockdown	2.52 (0.59 – 11.78)		
**Addiction**	Diagnosis of substance use and/or alcohol use disorder	0.99 (0.41 – 2.31)		
**Alcohol Use Disorder**	No	Ref		
	Yes	1.56 (0.40 – 5.83)		
**Social Support**	≤1 social supports	0.87 (0.32 – 2.22)		
**Resilience**	Low level of resilience	2.34 (1.09 – 5.05)	1.91 (0.80 – 4.63)	0.134
	Normal level of resilience	Ref	Ref	

Supplementary Table S4. Stressors reported by OPWH during first COVID-19 Lockdown in Kyiv

**Specific Stressors during lockdown**		**n (%)**
	Fear of getting infected with coronavirus	54 (43.9%)
	Movement restriction	46 (37.4%)
	No or few savings	35 (28.5%)
	Inflation, rising prices for things	33 (26.8%)
	Change of everyday social life	30 (24.4%)
	Loved ones' health	23 (18.7%)
	Receiving necessary healthcare services	20 (16.3%)
	Barriers to treatment for a chronic condition	18 (14.6%)
	Unemployment	16 (13.0%)
	Barriers to HIV treatment	12 (9.8%)
	Buying food, medicines, & other necessities	6 (4.9%)
	Surveillance by authorities	5 (4.1%)

Supplementary Table S5. Peer Support Preferences of OPWH in Kyiv

**Desired Characteristic**		**n (%)**
	HIV positive	59 (48.0%)
	Concordant gender	14 (11.4%
	Similar age	7 (5.7%)
	Concordant sexual orientation	4 (3.3%)
	Expertise in medicine	53 (43.1%)
**Willingness to provide Support**	Yes	72 (58.5%)
	No	51 (41.55)
